# Three-dimensional visualization of thyroid ultrasound images based on multi-scale features fusion and hierarchical attention

**DOI:** 10.1186/s12938-024-01215-1

**Published:** 2024-03-11

**Authors:** Junyu Mi, Rui Wang, Qian Feng, Lin Han, Yan Zhuang, Ke Chen, Zhong Chen, Zhan Hua, Yan luo, Jiangli Lin

**Affiliations:** 1 College of Biomedical Engineering, Sichuan University, Chengdu, Sichuan China; 2 China-Japan Friendship Hospital, Beijing, China; 3 Department of Ultrasound, General Hospital of Western Theater Command, Chengdu, Sichuan China; 4 Department of Ultrasound, West China Hospital of Sichuan University, Chengdu, Sichuan China; 5 Highong Intellimage Medical Technology (Tianjin) Co., Ltd, Tianjin, China

**Keywords:** Thyroid ultrasound video, Multi-target segmentation, 3D visualization, U-net++

## Abstract

**Background:**

Ultrasound three-dimensional visualization, a cutting-edge technology in medical imaging, enhances diagnostic accuracy by providing a more comprehensive and readable portrayal of anatomical structures compared to traditional two-dimensional ultrasound. Crucial to this visualization is the segmentation of multiple targets. However, challenges like noise interference, inaccurate boundaries, and difficulties in segmenting small structures exist in the multi-target segmentation of ultrasound images. This study, using neck ultrasound images, concentrates on researching multi-target segmentation methods for the thyroid and surrounding tissues.

**Method:**

We improved the Unet++ to propose PA-Unet++ to enhance the multi-target segmentation accuracy of the thyroid and its surrounding tissues by addressing ultrasound noise interference. This involves integrating multi-scale feature information using a pyramid pooling module to facilitate segmentation of structures of various sizes. Additionally, an attention gate mechanism is applied to each decoding layer to progressively highlight target tissues and suppress the impact of background pixels.

**Results:**

Video data obtained from 2D ultrasound thyroid serial scans served as the dataset for this paper.4600 images containing 23,000 annotated regions were divided into training and test sets at a ratio of 9:1, the results showed that: compared with the results of U-net++, the Dice of our model increased from 78.78% to 81.88% (+ 3.10%), the mIOU increased from 73.44% to 80.35% (+ 6.91%), and the PA index increased from 92.95% to 94.79% (+ 1.84%).

**Conclusions:**

Accurate segmentation is fundamental for various clinical applications, including disease diagnosis, treatment planning, and monitoring. This study will have a positive impact on the improvement of 3D visualization capabilities and clinical decision-making and research in the context of ultrasound image.

## Introduction

Ultrasound three-dimensional visualization holds significant importance in the field of medical imaging and is a highly promising cutting-edge technology. Traditional two-dimensional ultrasound images have limitations in displaying anatomical structures, while ultrasound three-dimensional visualization can present the morphology of organs and tissues in a more three-dimensional manner. This aids doctors in comprehensively understanding and identifying abnormalities, enhancing the readability of images, and improving the accuracy of clinical diagnosis. It possesses rich clinical applications and value.

Ultrasound (US) is radiation-free, inexpensive, not risk, real-time imaging and is frequently used to examine various diseases. However, two-dimensional ultrasound can be difficult to read, especially for novice doctors without clinical experience. Accurate interpretation of two-dimensional ultrasound often relies on the expertise of experienced clinicians. Therefore, it is crucial to develop three-dimensional visualization of ultrasound images to enhance their readability and facilitate interpretation by clinicians. Ultrasound three-dimensional visualization, a cutting-edge technology in medical imaging, enhances diagnostic accuracy by providing a more comprehensive and readable portrayal of anatomical structures compared to traditional two-dimensional ultrasound. In 2019, there were 567,233 cases of thyroid cancer worldwide, ranking it 9th in terms of incidence rate [1]. In China, a nationwide cross-sectional study conducted by the Chinese Society of Endocrinology and the Chinese Thyroid Association revealed that 20.43% of patients had thyroid nodules [2]. Thyroid diseases significantly impact human health. Therefore, the three-dimensional visualization method of ultrasound image is studied from the thyroid ultrasound image. This text describes a 3D visualization of the thyroid and surrounding tissues using a free arm ultrasound scanning video.

**Fig. 1:**
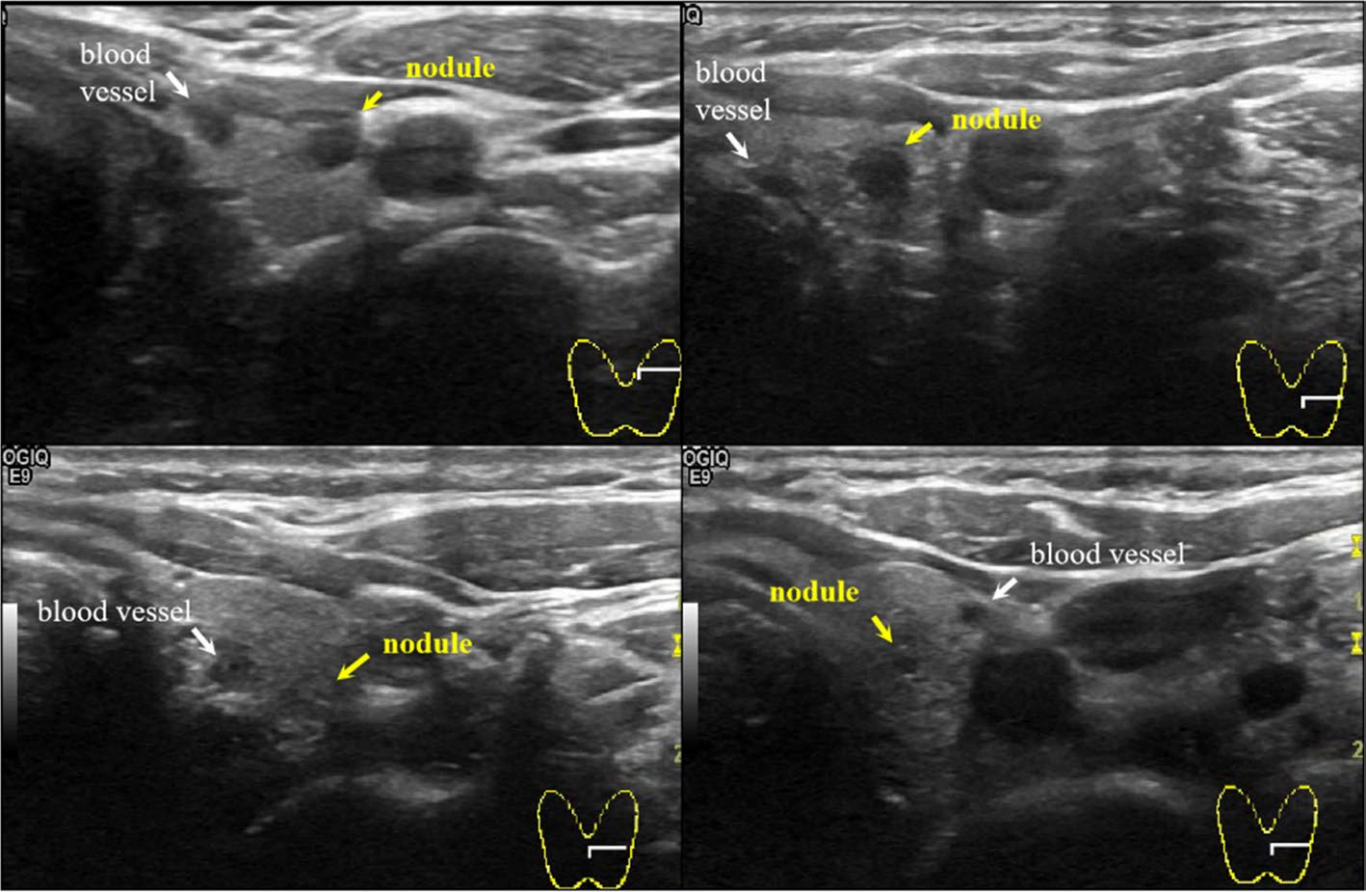
In thyroid ultrasound images, it is easy to get confused between vessels and nodules as they appear extremely similar

The key to achieving excellent 3D visualization of thyroid ultrasound images lies in accurate multi-target segmentation. However, the US is affected by speckle noise and echo perturbations, which make the image fuzzy and inhomogeneous. As shown in Fig. 1, there are many blood vessels interspersed in the thyroid gland, whose characteristics are often similar to those of nodules, and external vessels exhibit similar echogenic signals to the vesicles and the lesions in the US image [3]. In addition, the esophageal diverticula can invade the solid thyroid gland, with an echogenic appearance similar to nodules. And ultrasound imaging can be affected by the tumor microenvironment [4], as different microenvironments can result in varying tissue image representations. This can interfere with the recognition and segmentation of the target structure. The reasons for the appeal all lead to poor segmentation of multiple targets.

Previous research on multi-tissue segmentation of thyroid ultrasound images has shown that many studies struggle with accurately segmenting small targets and the background pixel blocks are wrongly segmented. To enhance the segmentation effectiveness of multiple tissues surrounding the thyroid, this work proposed a framework called PA-Unet++. PA-Unet++ improves upon the U-net++ architecture by incorporating two key components: the pyramid pooling module (PPM) and attention gating (AG). The addition of PPM allows for an expanded receptive field within the network, enabling the integration of multi-scale features and global context information. This integration enhances the network’s capability to accurately segment target structures at various scales. Furthermore, the AG mechanism was adopted to strengthen the network’s attention to the region of interest and to highlight the target organizational structure while reducing the influence of the background. This attention mechanism ultimately enhanced the overall segmentation effect for all structures.

Using the aforementioned algorithm, accurate segmentation of multiple tissues, including the thyroid, nodules, and internal thyroid blood vessels, was achieved, and then utilized for 3D visualization. The 3D visualization results in a clearer distinction between thyroid nodules and invasive lesions of blood vessels and other tissues within the thyroid gland, leading to a more precise diagnosis of esophageal diverticulum. Moreover, the intuitive spatial location information can serve for treatment planning and surgical navigation.

## Related works

According to the currently retrieved research, many models, such as U-net, ACU2E-net, BPAT-UNet, FCG-net, SK UNet++, etc. [5–10], were used in the form of encoding and decoding for target segmentation of nodules or entities in ultrasound thyroid. As displayed in Table 1, Chen et al. [6] combined U-net with traditional algorithms to obtain the original data, and super-pixel processed data and Sobel edge processed images were merged as the training data as a complement to enhance the segmentation of thyroid entities. Bi et al. [7] applied the boundary point supervision module and adaptive multi-scale feature fusion module to transformer U-Net to improve the boundary segmentation effect of nodules with small nodule segmentation. Shao et al. [8] proposed FCG-Net by replacing the encoder and decoder with GB module based on the full-scale jump connection of Unet3 + as a way to improve nodule segmentation. Dai et al. [9] proposed FCG-Net based on U-net++ by replacing every block with SK modules and eliminating some of the skip-connections to achieve segmentation of nodules; Balachandran et al. [10] replaced each block in U-net by a separate attention mechanism U-net with different depths and proposed ACU2E-Net to segment thyroid entities.

**Table 1:** The related research

Parts	Authors	Methods	Deficiencies
Thyroid	Chen (2023)	Unet + sobel	Need to do a lot of preorder calculations to get training inputs
Thyroid	Balachandran (2023)	ACU2E-Net	Huge computation
Nodules	Bi (2023)	BPAT-UNet	Huge computation
Nodules	Shao (2023)	FCG-Net	Not much improved compared with the basic model
Nodules	Dai (2023)	SK-Unet++	Higher image quality requirements, large error distance for mis-segmentation
Multi-target	Kumar (2020)	MPCNN	Not good for small nodules with internal vesicles
Multi-target	Webb (2020)	DeepLabv3 + LSTM	Poor segmentation results for nodules
Multi-target	Luo (2021)	Cascade R-CNN	No segmentation of nodules, internal vessels, poor segmentation results for small targets
Multi-target	Ma (2022)	SPRMaskR-CNN	Detection identification and segmentation is done in two steps. Cannot identify some quite small organs
Multi-target	Zheng (2023)	DSRU-Net	Poor segmentation of small nodules

For the task of single-target segmentation, only one target needs to be optimized. However, in the case of multi-target segmentation, improving one sub-target may lead to a decrease in performance of another or several other sub-targets. Therefore, the model needs to coordinate among multiple objectives to achieve a common optimal solution. Additionally, compared to single-target segmentation, multi-target segmentation requires more accurate feature extraction and discrimination of each target. Therefore, in the task of multi-target segmentation, the model will usually strengthen the capture and distinction of each target feature. To improve the segmentation effect of different targets.

For multi-target segmentation of thyroid ultrasound images, Kumar et al. [11] proposed a framework for the simultaneous segmentation of thyroid, thyroid nodules, and thyroid follicles, but it is less effective in segmenting smaller internal nodules and vesicles. Webb et al. [12] used the feature results from six DeepLabv3 + outputs as sequence inputs to the LSTM for loop training, combined with spatial pyramid pooling, to obtain the final segmentation results for thyroid solids, nodules, and vesicles. Similarly, the problem of poor segmentation of smaller internal nodules and vesicles was not resolved. Luo et al. [13] proposed cascade R-CNN, which combines object detection with semantic segmentation network to segment anterior cervical muscle, cricoid cartilage, trachea, thyroid, blood vessels, and esophagus simultaneously. Ma et al. [14] introduced ROI Align in the segmentation head part based on Mask R-CNN to generate and combine multi-scale feature information for segmenting the right and left lobes of the thyroid gland, isthmus, muscle, trachea, carotid artery, jugular vein, esophagus, and cricoid cartilage with the internal vascularity of the thyroid gland. Both of their models are less effective at segmenting smaller organizational structures, with the worst AP only reaching 27.8% (endothyroid vessels). Zheng et al. [15] proposed deformable-pyramid split-attention residual U-Net (DSRU-Net) by introducing ResNeSt block, atrous spatial pyramid pooling, and deformable convolution v3 based on U-Net. It was used to segment the thyroid solids and nodules. However, the segmentation of smaller nodes is not as effective as larger entities.

For 3D visualization of the thyroid gland, Thiering et al. [16] developed a method for a high-resolution 3D reconstruction of the thyroid from two-dimensional ultrasound data stacks based on its data fusion with CT images. Poudel et al. [17] segmented thyroid images in 703 images and passed them to a 3D reconstruction algorithm to obtain a 3D model of the thyroid. Ciora et al. [18] achieved a technique for the thyroid 3D model reconstruction from 2D images provided by an ultrasound system using image processing and pattern recognition. Wein et al. [19] proposed a framework for deep learning-based trajectory estimation of overlapping horizontal and sagittal image data to assist in 3D model optimization. However, the existing three-dimensional reconstruction is aimed at the thyroid, not the thyroid nodules and its surrounding tissue structure. It is only capable of representing the external contours of the thyroid entity and does not characterize the internal structure. Moreover, it cannot characterize the spatial information between different tissues.

While some advanced segmentation algorithms for thyroid ultrasound images have performed well, there are still issues with inaccurate segmentation of small targets and incorrect segmentation of background pixel blocks. Additionally, existing three-dimensional visualizations only focus on the thyroid entity, neglecting other tissues. It is important to address these limitations in future research. Therefore, this paper proposes PA UNET++ to enhance the segmentation of multiple targets and uses the segmentation results of thyroid ultrasound multi-tissue to achieve three-dimensional visualization. This visualization clearly represents the various tissues and their spatial relationships.

## Materials and methods

### Data

The data used in this study were obtained from 200 desensitized thyroid ultrasound scan videos. Two-dimensional image data were obtained by intercepting from the videos, some of which contained thyroid nodules and some of which did not. The videos were obtained by an experienced sonographer who performed a top-to-bottom transverse scan of the left or right lobe of the thyroid gland. The videos were clear and included target tissues such as the thyroid, trachea, esophagus, and carotid arteries, as required for this study. The ultrasound machine is a GE model Versana Premier Pt, the probe is a GE12L-RS, and the sampling frequency is 8–10 MHz. All images were preprocessed to remove sensitive letters. Interference from interface markers was excluded and ROI regions were extracted. Finally, 4600 images with more than 23,000 annotated regions were obtained, which were divided into a training set and a test set in a ratio of 9:1.

**Fig. 2:**
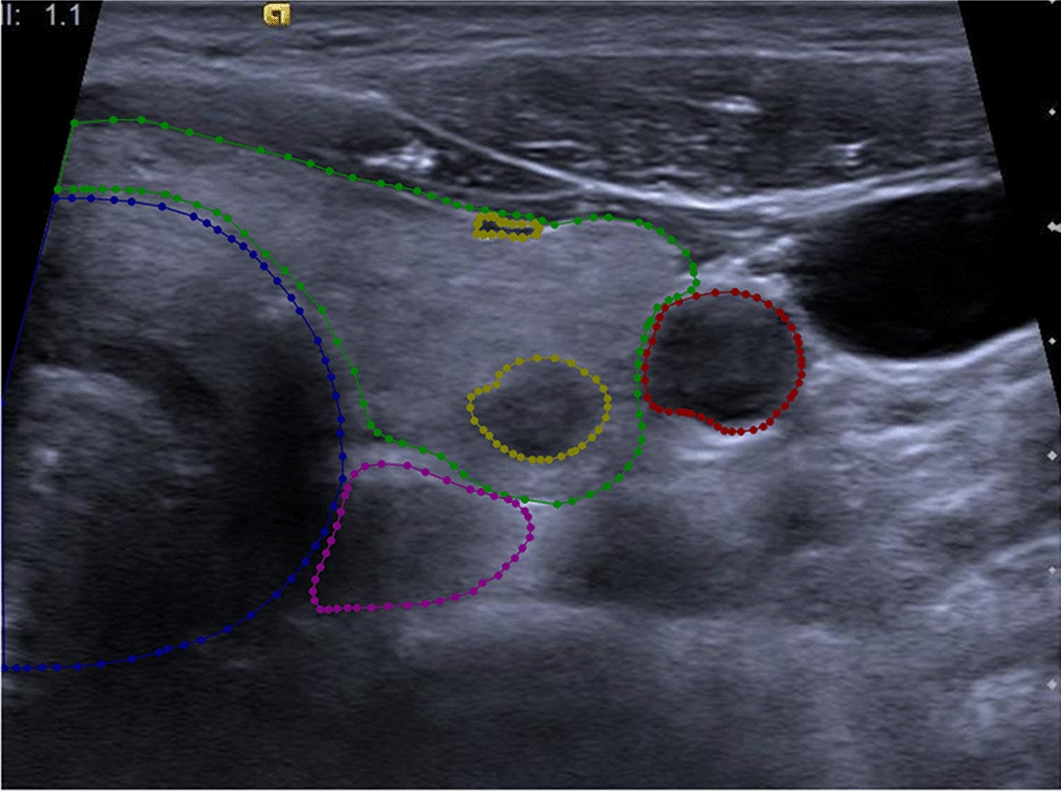
The ground truth of different organizational structures

The labeled images depict various anatomical structures such as the thyroid, trachea, esophagus, blood vessels, as well as thyroid nodules (including vessels in the thyroid gland), as illustrated in Fig. 2. The red circle denotes blood vessels (carotid artery), the blue circle represents the trachea, the green border depicts the thyroid, the yellow circle indicates nodules or vessels (NoV) in the thyroid, and the purple circle signifies the esophagus. The labeling process was supervised by experienced ultrasound doctors, and the final results were reviewed by a professional ultrasound doctor with extensive clinical expertise. Any incorrect labels were promptly corrected. Before segmentation, denoising is usually required. Traditional denoising filters and deep learning denoising models, such as RED-MAM, LPRNN, CS Net, etc., are helpful for thyroid segmentation tasks [20–24]. The training data are also enhanced through random flipping, rotation, cropping, scaling, and other methods.

### PA-Unet++ model

**Fig. 3:**
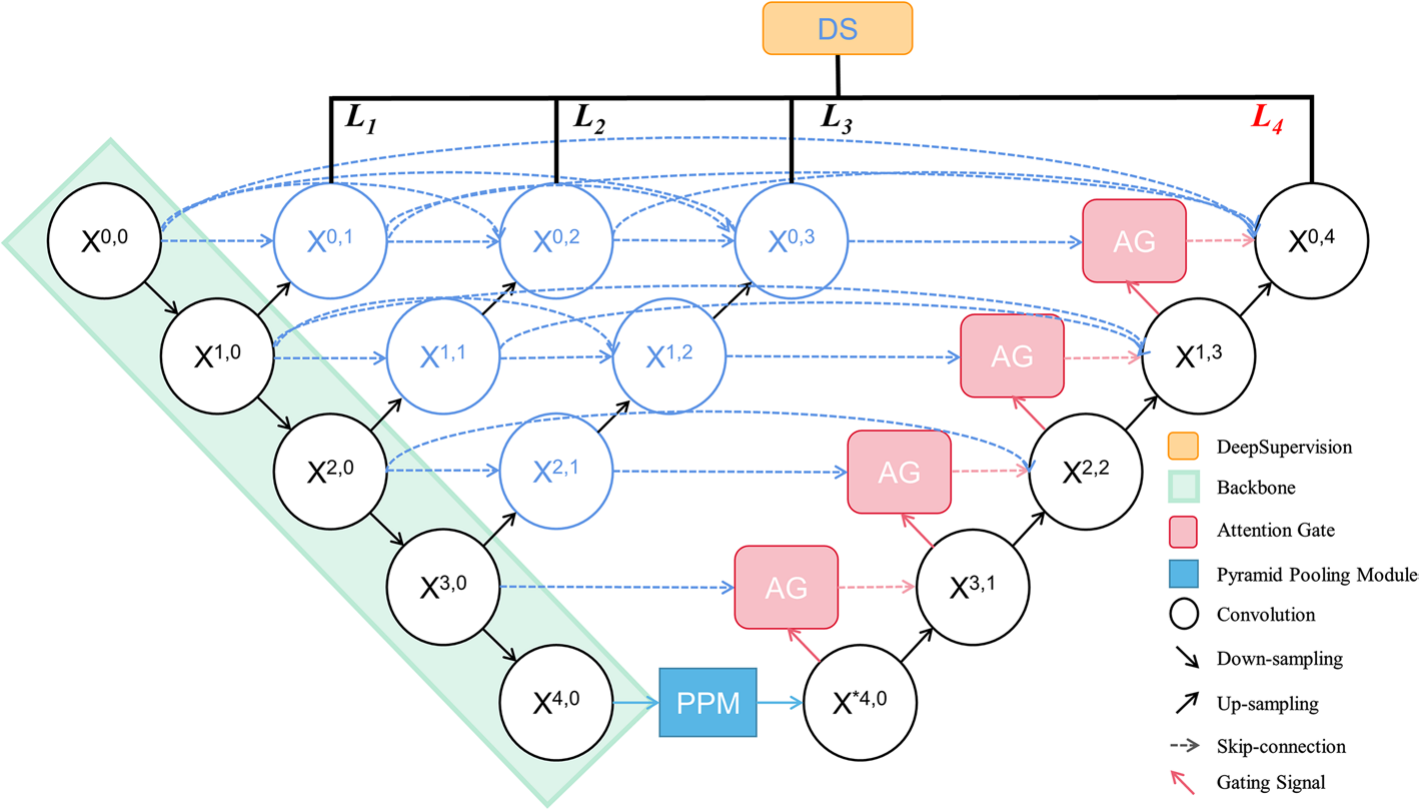
The deepest feature layer generated in the coding phase of the model is sent to the PPM module to collect the local information and global context information carried by different sub-regions. In the decoding phase, the AG attention mechanism is used to improve the target region of interest (ROI) weight

The U-net++ model uses dense skip-connections to combine context information and multi-scale feature information. However, this approach can result in a loss of edge features and spatial information in the image. Thus, in order to expand the network's receptive field again, gradually enhance the network's attention to the target area, and strengthen the capture of edge features and spatial information, we proposed PA-Unet++ which is shown in Fig. 3. Pyramid pooling module (PPM) and attention gating (AG) are introduced into the U-net++ model. In view of the requirements of multi-classification semantic segmentation in this task, and thyroid ultrasound images have special image characteristics such as high noise, and low contrast, Lovasz-Softmax loss is used as the loss function of this model in the process of network optimization training.

### Pyramid pooling module

In convolution neural networks, the size of the receptive field can roughly indicate the amount of context information used by the network. Zhou et al. [25] showed that the empirical receptive domain of convolution neural networks is much smaller than the theoretical receptive domain, especially in high-level feature extraction, which makes many of the models not fully integrated into important global scenarios. An effective global prior module is proposed to solve this issue. Global average pooling as a global context prior is a good baseline model, but for the complex scene image of ultrasound thyroid, this strategy is not enough to cover the necessary information. The pixels in these ultrasound images are labeled for many structures and tissues. If they are fused directly to form a single feature vector, the spatial relationship may be lost, resulting in ambiguity. The global context information and the context information of sub-regions help to distinguish various structural categories in this regard. A more powerful algorithm can fully integrate information from different sub-regions with these receptive regions. To further reduce the loss of context information between different sub-regions, a multi-level global priori is proposed, which includes context information of different scales and changes between different sub-regions.

**Fig. 4:**
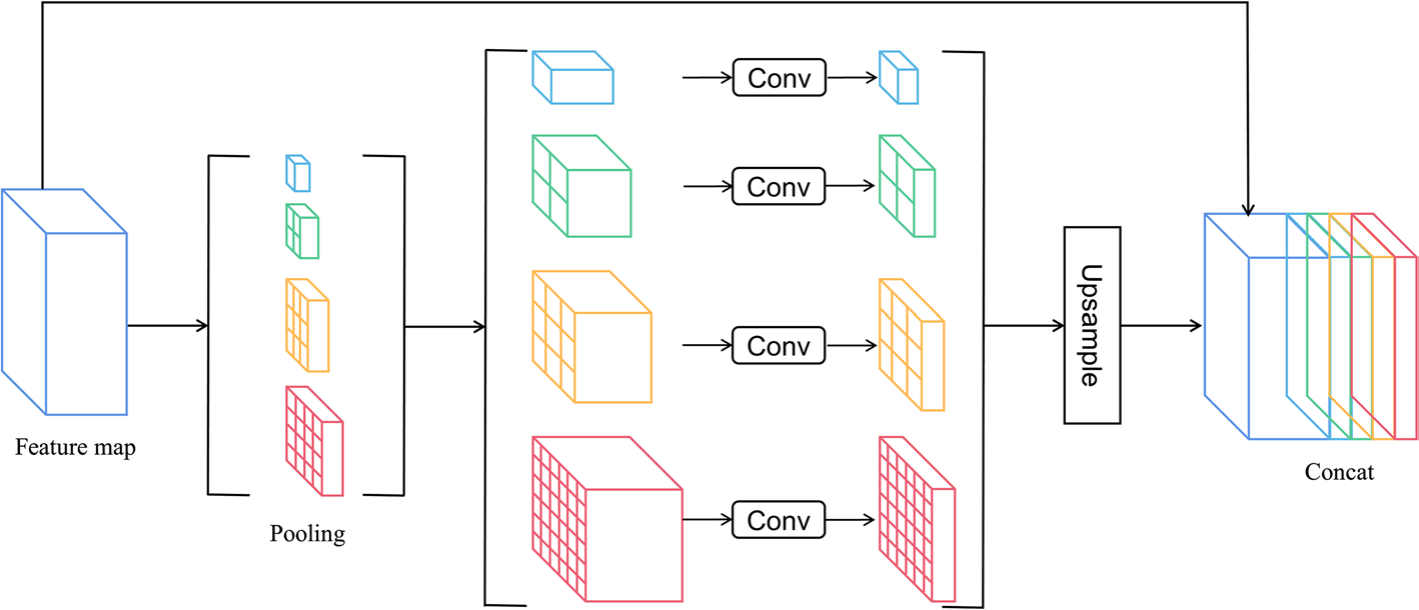
The deepest feature layer is pooled with different kernel sizes, then the pooling result is adjusted channels and up-sampled through convolution. Finally, all the feature layers obtained are connected with the original input features to form the final feature representation

In Fig. 4, the pyramid pooling module [26] fuses features at four different pyramid scales. The pyramid layer below divides the feature maps into different sub-regions and forms a collective representation of different positions. Calculated by Eq. (1), different levels of output in the pyramid pool module contain feature maps with different sizes. In order to maintain the weight of global features, if the horizontal size of the pyramid is *N*, one $1\times 1$ convolution layer is used after each pyramid level to reduce the dimension of context representation to 1/N of the original representation. Then, the low-dimensional feature map is directly up-sampled by bilinear interpolation to obtain features of the same size as the original feature map. Finally, the features at different levels are connected to the final global features of the pyramid pool. This structure abstracts different sub-regions by using pooled kernels of different sizes. Therefore, the multi-phase kernel should maintain a reasonable gap in presentation:

1 $$\begin{array}{l}\left\{\begin{array}{c}{O}_{{\text{H}}}=\frac{{I}_{{\text{H}}}-{K}_{{\text{H}}}}{S}+1\\ {O}_{{\text{W}}}=\frac{{I}_{{\text{W}}}-{K}_{{\text{W}}}}{S}+1\end{array}\right.,\end{array}$$

where the ${I}_{{\text{H}}}$, ${I}_{{\text{W}}}$ denote the height and width of the input feature map, ${K}_{{\text{H}}}$, ${K}_{{\text{W}}}$ denotes the height and width of the pooling kernel, and $S$ denotes the step size of the pooling kernel, ${O}_{{\text{H}}}$, ${O}_{{\text{W}}}$ denote the height and width of the output feature map.

### Attention gate

**Fig. 5:**
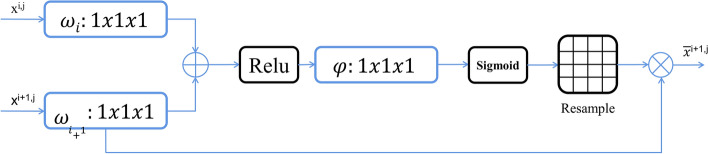
The feature layers of the upper layer and the current layer are used as inputs to enter the attention mechanism gating, and the attention coefficient is calculated to weigh the current feature layer for decoding

In order to capture a receptive field large enough to obtain semantic context information, the feature map grid is gradually down-sampled in the standard CNN architecture. In this way, rough spatial grid-level features can simulate the position and relationship between organizations in the global scope. However, for small objects with large shape variability, it is still difficult to reduce false positives. To improve accuracy, the current segmentation framework [27–29] relies on additional previous object positioning models to simplify tasks into separate positioning and subsequent segmentation steps. This goal can be achieved by integrating attention gating (AG) in a standard CNN model. This avoids the need for extensive model training and additional model parameter increments. Compared with the multi-stage CNN positioning model, AG gradually suppresses the feature response of irrelevant background regions without cutting ROI between networks. As displayed in Fig. 5, the characteristic graph of the encoding part of the previous layer and the decoding part of the current layer are used as the input of AG. In AG, the two parts are added after being processed by $1\times 1$ convolution layer and batch normalization (BN) in parallel, and the channel is adjusted by $1\times 1$ convolution layer and BN layer after being operated by Relu, then Sigmoid activation is implemented. The feature information of which linear and nonlinear transformation is completed. And calculated by Eq. (2), the attention coefficient (weight) is generated through the resampling step, and it is multiplied with the current decoding feature map. The AG result is connected and fused with the feature map of the upsampling decoding part, the AG at the next level is used as the input to participate in the decoding part of the whole network, so as to continuously improve the weight of the target ROI and suppress the non-ROI part.

2 $$\begin{array}{c}{Q}_{{\text{att}}}={\sigma }_{2}\varphi \left({\sigma }_{1}\left({\omega }_{i}{x}_{i}+{\omega }_{i+1}{x}_{i+1}+{b}_{1}\right)\right)+{b}_{2},\end{array}$$

where the ${Q}_{{\text{att}}}$ is the attention coefficient, $\varphi$, ${\omega }_{i}$, ${\omega }_{i+1}$ are the convolution operation, and ${\sigma }_{1}$ denotes the ReLU, and ${\sigma }_{2}$ denotes the Sigmoid, ${b}_{1}$, ${b}_{2}$,are the bias term corresponding to the convolution.

### Lovasz-Softmax loss

A good performance indicator for evaluating the segmentation mask, usually used in semantic segmentation models, is the Jaccard [30] index, also known as the intersection over union (IOU). Given the ground truth vector $Y$ and the predicted vector ${Y}^{*}$, the Jackard index of class $c$ is defined as:

3 $$\begin{array}{c}{J}_{{\text{c}}} \left(Y,{Y}^{*}\right)=\frac{\left|\left\{Y=c\right\}\cap \left\{{Y}^{*}=c\right\}\right|}{\left|\left\{Y=c\right\}\cup \left\{{Y}^{*}=c\right\}\right|}.\end{array}$$

The ratio of the true mask and the calculated mask on their union is [0,1], and the convention is 0/0 = 1. The corresponding loss function used in empirical risk minimization is:

4 $$\begin{array}{c}{\Delta }_{{J}_{c}}\left(Y,{Y}^{*}\right)=1-{J}_{c}\left(Y,{Y}^{*}\right).\end{array}$$

For multi-label datasets, the Jaccard index is usually averaged across classes to produce a mean IoU (mIoU). For split output ${Y}^{*}$ and ground truth $Y$, define the error prediction pixel set of class $c$ as:

5 $$ E_{c} (Y,\;Y^{*} ) = \{ Y = c,\;Y^{*} \ne c\} \cup \{ Y \ne c,\;Y^{*} = c\} . $$

For a fixed ground truth $Y$, in Eq. (4) Jaccard loss can be modified to another function with incorrect prediction:

6 $$\begin{array}{c}{\Delta }_{{J}_{c}} : {E}_{c}\in {\left\{\mathrm{0,1}\right\}}^{p} \mapsto \frac{\left|{E}_{c}\right|}{\left|\left\{Y=c\right\}{\cup E}_{c}\right|},\end{array}$$

where the $p$ is the number of pixels in the concerned image or small batch processing. The indicator vector in the discrete hypercube ${\left\{\mathrm{0,1}\right\}}^{p}$ is used to identify the subset of pixels. The Jaccard loss is only applicable to discrete space. That means, when the input is 0 or 1, it will cause the problem of non-derivative in continuous space. If the network prediction result is continuous, the discretization will lead to non-derivative and cannot be directly connected behind the network. Therefore, it is desired to assign the loss to any error vector $E$ in the continuous optimization settings ${E}_{c}\in {\mathbb{R}}_{+}^{p}$ and not just a discrete vector that is incorrectly predicted in ${\left\{\mathrm{0,1}\right\}}^{p}$. In general, the convex closure of a set function is np-hard. Moreover, the Jaccard set functions have been proven to satisfy the properties of submodular functions [31].

Definition of submodule function [32]: for a set function $\Delta :{\left\{\mathrm{0,1}\right\}}^{p}\to {\mathbb{R}}$ for all A, B$\in {\left\{\mathrm{0,1}\right\}}^{p}$:

7 $$\begin{array}{c}\Delta \left(A\right)+\Delta \left(B\right)\ge \Delta \left(A\cup B\right)+\Delta \left(A\cap B\right).\end{array}$$

Thus, a Lovasz extension is performed on the Jaccard loss to extend the input discrete space ${\left\{\mathrm{0,1}\right\}}^{p}$ to the entire continuous ${\mathbb{R}}^{p}$. Its output value is equal to the output value of the original function on ${\left\{\mathrm{0,1}\right\}}^{p}$ has convexity, and the optimization direction is consistent. For a set function $\Delta :{\left\{\mathrm{0,1}\right\}}^{p}\to {\mathbb{R}}$ and satisfying $\Delta (0)$ = $0$, the lovasz extension [33] is defined as:

8 $$ \overline{\Delta }:\;E \in {\mathbb{R}}^{p} \mapsto \sum\nolimits_{i = 1}^{p} {E_{i} } g_{i} (E), $$

and:

9 $$\begin{array}{c}{g}_{i}\left(E\right)=\Delta \left(\left\{{\pi }_{1},\cdots {,\pi }_{i}\right\}\right)-\Delta \left(\left\{{\pi }_{1},\cdots {,\pi }_{i-1}\right\}\right),\end{array}$$

$\pi$ is to arrange the components of $E$ in descending order, i.e., ${x}_{\pi 1}\ge {x}_{\pi 2}\cdots \ge {x}_{\pi p}$.

Let $$\Delta $$ be the set function of coding submodule loss, such as the defined Jaccard loss. By submodulation, $$\overline{\Delta  }$$ is a compact convex closure of $$\Delta $$. $$\overline{\Delta  }$$ is piecewise linear, and in any error prediction set $${E}_{c}$$, the interpolation value of $$\Delta $$ in $${\mathbb{R}}^{p}$$\$${\left\{\mathrm{0,1}\right\}}^{p}$$, have the same value as $$\Delta $$ in $${\left\{\mathrm{0,1}\right\}}^{p}$$. Intuitively, if $$E$$ is the vector of all pixel errors, then $$\overline{\Delta  }(E)$$ is the sum of these errors weighted according to the interpolation discrete loss. Due to its convexity and continuity, $$\overline{\Delta  }$$ is a natural alternative to minimization $$\Delta $$ using first-order continuous optimization. For example, in the current deep learning framework, calculate $$\overline{\Delta  }$$ that basic operations involved in (sorting, dot product, …) are differentiable and implemented on the GPU. The vector $$g(E)$$, whose component is defined in Eq. (9), directly corresponds to $$\overline{\Delta  }$$ the derivative with respect to $$E$$.

$c\in C$ is the object class $c$ in the total class number $C$, ${f}_{i}\left(c\right)$ is a vector of the network output. Assume that the non-normalized score ${{\text{F}}}_{{\text{i}}}\left({\text{c}}\right)$ of the network has been mapped to through the Softmax unit and the probability is:

10 $$\begin{array}{c}{f}_{i}\left(c\right)=\frac{{e}^{{F}_{i}\left(c\right)}}{{\sum }_{{c}^{\mathrm{^{\prime}}}\in C}{e}^{{F}_{i}\left({c}^{\mathrm{^{\prime}}}\right)}} \quad \forall i\in \left[1,p\right],\forall c\in C, \end{array}$$

hence, the Lovasz extension is combined with Softmax loss, and the object class probability ${{{f}}}_{{{i}}}\left({\text{c}}\right)\in \left[\mathrm{0,1}\right]$ of Eq. (3–10) a is used to construct the pixel error vector $E\left(c\right)$ of $c\in C$:

11 $$\begin{array}{l}{E}_{i}\left(c\right)=\left\{\begin{array}{l}1-{f}_{i}\left(c\right) \quad \,  {\text{if}}\,  c={Y}_{i}^{*}\\ {f}_{i}\left(c\right) \qquad \quad {\text{otherwise}} \end{array}\right..\end{array}$$

Using the pixel error vector $E\left(c\right)$ to construct alternative ${\Delta }_{Jc}$. For object class $c$, the Jaccard index is:

12 $$\begin{array}{c}{\text{loss}}\left({\varvec{f}}\left(c\right)\right)=\overline{{\Delta  }_{{J}_{c}}}\left(E\left(c\right)\right).\end{array}$$

Considering the common class average mIOU measurement in semantic segmentation, the average of specific classes is replaced; Then, Lovasz-Softmax loss [34] is defined as:

13 $$\begin{array}{c}{\text{loss}}\left({\varvec{f}}\right)=\frac{1}{\left|C\right|}\sum_{c\in C}\overline{{\Delta  }_{{J}_{c}}}\left(E\left(c\right)\right).\end{array}$$

This loss function is an optimization of IOU loss. In the continuous optimization scheme, each component of the error vector is allocated and optimized. It performs better than cross-entropy loss in multi-classification semantic segmentation. It is suitable for the requirements of multi-structure segmentation in thyroid ultrasound images with high noise, low contrast, and very similar characterization of different tissue structures in this task.

## Results

### Evaluation index

Since the semantic segmentation in this task is essentially another type of pixel-level classification. To assess the performance of the segmentation model, we employed a confusion matrix.

The dice coefficient is a set similarity measurement function, which is usually used to calculate the similarity between two sets. It can be used to calculate the similarity between the prediction result and the true label in the semantic segmentation task to evaluate the segmentation effect. The dice coefficient is defined by the confusion matrix as:

14 $$\begin{array}{c}{\text{Dice}}=\frac{2{\text{TP}}}{2{\text{TP}}+{\text{FP}}+{\text{FN}}},\end{array}$$

where ${\text{TP}}$: positive samples predicted by the model to be in the positive category, ${\text{TN}}$: negative sample predicted by the model to be in the negative category, ${\text{FP}}$: negative sample predicted by the model to be in the positive category,${\text{FN}}$: positive samples predicted by the model to be in the negative category.

Intersection over union (IOU) is the ratio of the intersection and union of the predicted result of a certain category and the true label. The IOU is defined as:

15 $$\begin{array}{c}{\text{IOU}}=\frac{{\text{TP}}}{{\text{TP}}+{\text{FP}}+{\text{FN}}}.\end{array}$$

For multi-category semantic segmentation, the average intersection over union ratio mean IOU (mIOU) is generally used as the evaluation indicator, that is, the IOU of each category is summed and then averaged.

Pixel accuracy (PA), the percentage of correct predicted pixels in the total number of pixels. The PA is defined as:

16 $$\begin{array}{c}{\text{PA}}=\frac{{\text{TP}}+{\text{TN}}}{{\text{TP}}+{\text{FP}}+{\text{TN}}+{\text{FN}}},\end{array}$$

category pixel accuracy (CPA), the percentage of pixels whose real tags also belong to category $c$ among all pixels whose prediction result is category $c$. The CPA is defined as:

17 $$ {\text{CPA}} = \frac{{{\text{TP}}}}{{{\text{TP}} + {\text{FP}}}}. $$

Thereby, the above evaluation indicators are used in this task to evaluate the segmentation effectiveness of the model.

### The results of multi-organization segmentation

**Fig. 6:**
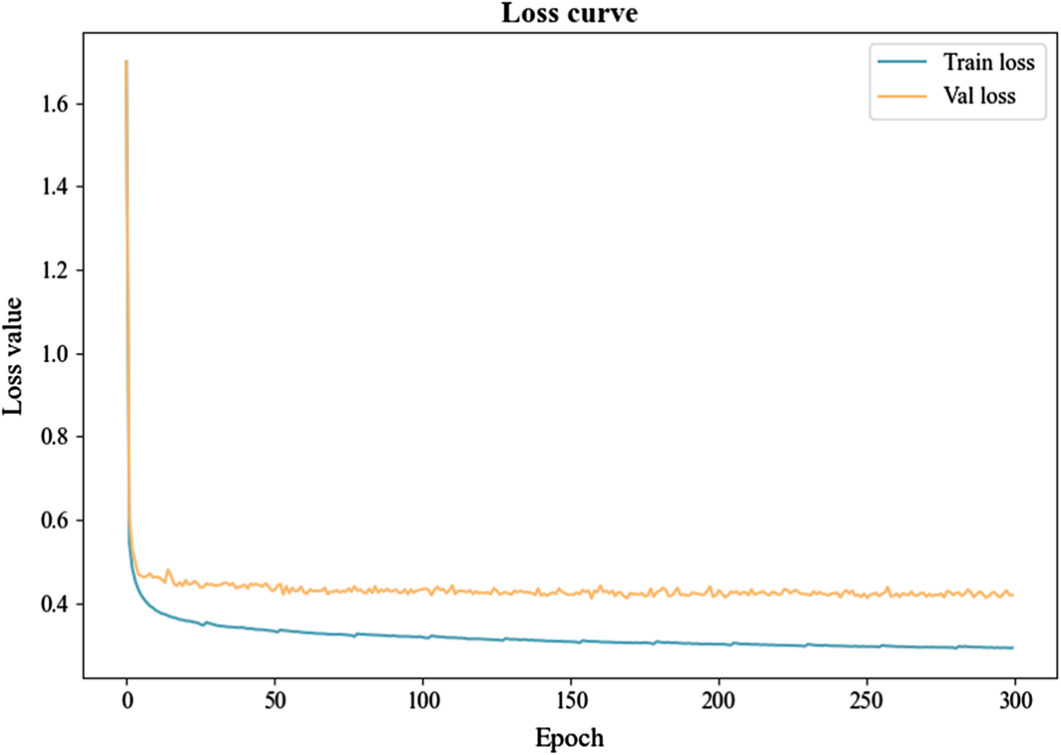
The loss curves of the training phase

The improved U-net++ network model was constructed using the PyTorch framework, with a learning rate of 0.0001. The learning rate decayed to 0.9 of its original value at the 100th and 150th epochs, as per external demand. The Adam optimizer was used throughout the training process with a potential energy of 0.9, and the batch size was set to 16. Prior to input into the model, all images were resized to 256 × 256. The training was conducted on a server with a GPU of NVIDIA GeForce RTX 3090 × 0.5 and 24G memory. As shown in Fig. 6, it is the loss curve of the training of the model, you can see that the model converges faster and tends to be stable.

Based on the algorithms mentioned in the previous method, the following experimental verifications are present:

The improved U-net++ network model includes a dense skip connection that enables the combination of upper-layer features during decoding. Additionally, the use of deep supervision (DS) allows for decoding from different feature layers or a combination of all feature layers. By determining the optimal network depth for the training data, we were able to conduct comparative experiments for the four decoding methods. Specifically, L2–L4 represent decoding at three depths of U-net++. Additionally, the original image undergoes denoising through Non-local mean (NLM) filtering. A comparison of the training before and after denoising is conducted based on DS. The resulting segmentation is also evaluated. The model was trained for 300 epochs, and the Dice coefficient, mIOU, and PA effects were evaluated.

As shown in Table 2, deep supervision (DS) outperforms the other decoding methods in all three evaluation indexes of segmentation results. Specifically, the DS with NLM get a better result than DS, the Dice is 0.7933, the mIOU is 0.7451 and the PA is 0.9315. It is evident that denoising can improve segmentation performance to some extent. Compared with others, the DS significantly improves the Dice, mIOU, and PA indexes. In traditional feature extraction, feature maps are continuously sampled and compressed, leading to the inevitable loss of relevant information. This makes it difficult for the network to retain and pay attention to the boundary shape information of the organizational structure during the continuous in-depth feature extraction, resulting in a significant loss for the semantic segmentation task. Therefore, the utilization of the deep supervision (DS) algorithm enables the combination of multi-scale context feature information which is particularly important for multi-organization segmentation tasks as shallow features can retain spatial location information of various organizational structures.

**Table 2:** Comparison experiment of decoding methods

Decode	Dice	mIOU	PA
L2	0.6841	0.5439	0.8704
L3	0.7585	0.6697	0.9147
L4	0.7878	0.7344	0.9295
DS	0.7905	0.7373	0.9306
DS (NLM)	**0.7933**	**0.7451**	**0.9315**

The Dice (dice coefficient) is a set similarity measurement function, which is usually used to calculate the similarity between two sets. You can see in Eq. 14

The IOU (intersection over union) is the ratio of the intersection and union of the predicted result of a certain category and the true label. You can see in Eq. 15

For multi-category semantic segmentation, the average intersection over union ratio mean IOU (mIOU) is generally used as the evaluation indicator, that is, the IOU of each category is summed and then averaged

The PA (pixel accuracy) is the percentage of correct predicted pixels in the total number of pixels. You can see in Eq. 16

The CPA (category pixel accuracy) is the percentage of pixels whose real tags also belong to category. You can see in Eq. 17

**Fig. 7:**
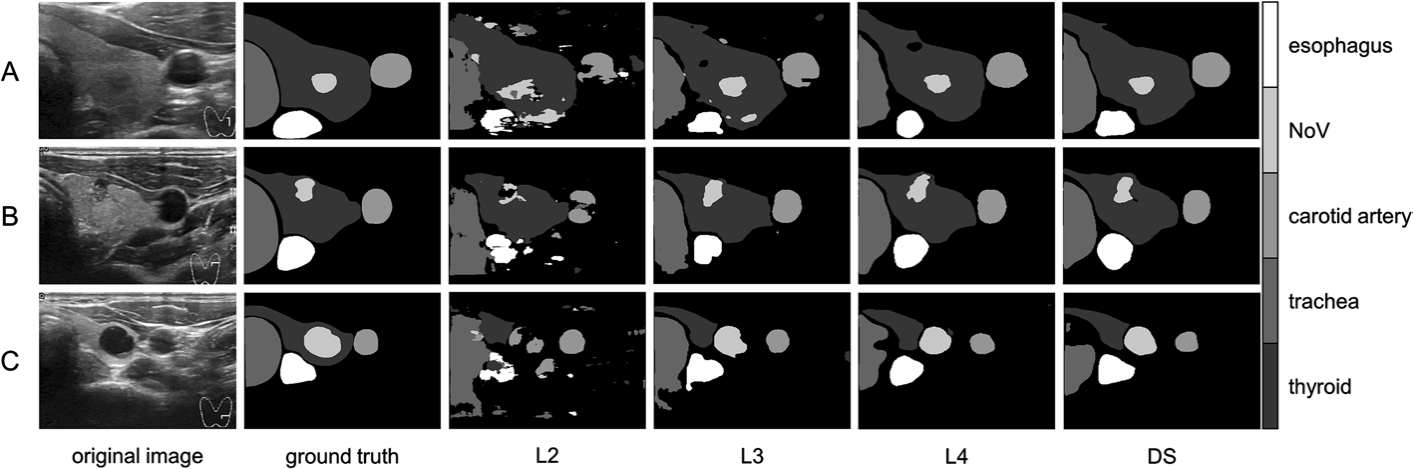
Segmentation results with different decoding methods

In Fig. 7, it can be observed that, as the network deepens and dense skip-connections increase, along with enhanced feature extraction, contextual information combination, and global information representation capabilities, the segmentation results from L2 to DS exhibit a gradual improvement. Although the ideal effect has not been achieved, five target structures are basically located and segmented. L2 is located shallowly in the network, which results in a larger extracted feature map and a greater amount of spatial positional information retained. However, it lacks sufficient learning and extraction of texture information unique to the target structure. Therefore, its segmentation results can only broadly represent the spatial positional relationships between the target structures, without being able to segment and represent the specific boundaries of each structure. As the network depth increases, so does its ability to extract features, while the contextual information associated with skip-connections becomes more comprehensive. As a result, the segmentation results of L3–L4 exhibit a significant improvement, with more accurate positioning of the target structure and clearer boundaries, albeit with poor boundary integrity. Ultimately, the DS method yields significantly improved segmentation results, with precise positioning of spatial positional relationships between various organizational structures, as well as enhanced boundary integrity and shape accuracy for each structure.

**Fig. 8:**
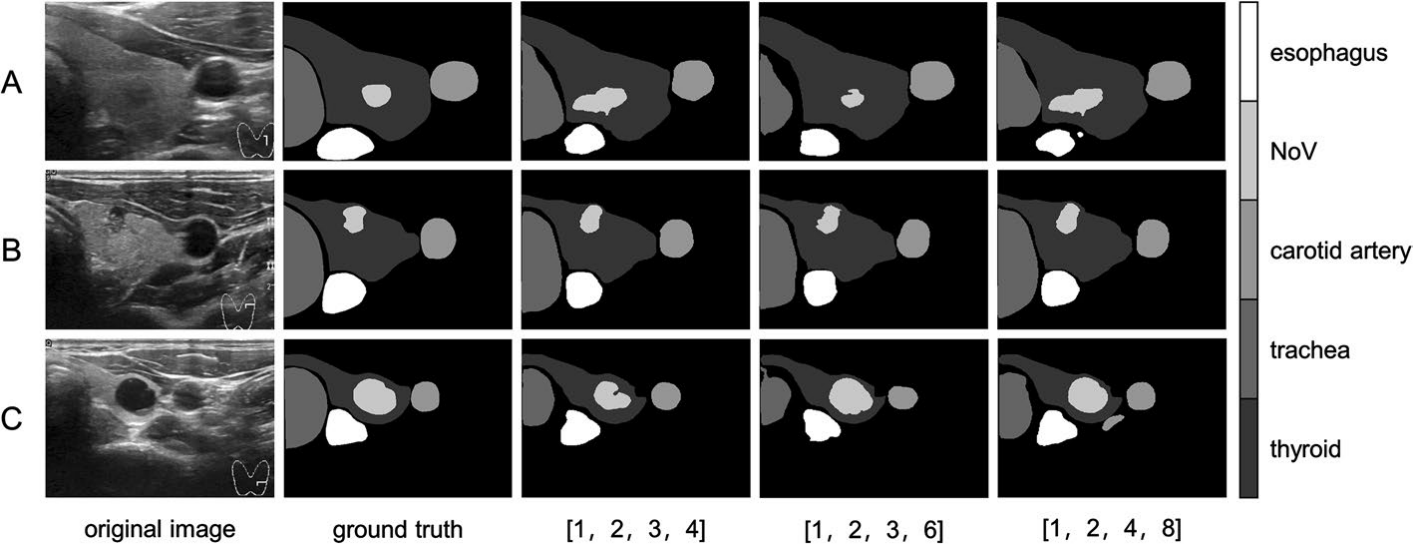
Segmentation results with different combinations of pyramid pooling kernel sizes

To fuse context information containing changes between different scales and sub-regions, the PPM module employs feature fusion at four different pyramid scales. The pyramid layer divides the feature map into various sub-regions and forms a set representation of different positions, abstracting different sub-regions using pooled kernels of varying sizes. Finally, features at different levels are connected to yield the final pyramid of pooled global features. To determine the appropriate pooled kernel size, we designed an experimental control group, conducting experiments with pool cores set to (1, 2, 3, 4), (1, 2, 3, 6), and (1, 2, 4, 8). We trained the model for 300 epochs under these three parameter settings and evaluated the Dice coefficient, mIOU, and PA effects:

Table 3 demonstrates that the pooling kernels set as (1, 2, 3, 6) yield relatively more favorable outcomes, with a Dice score of 0.8087 and an mIOU of 0.7887. Moreover, the pooling kernels set as (1, 2, 4, 8) produce the highest PA index of 0.9446. When dealing with the deepest feature map with a small size, pooling kernel size combinations with excessively small intervals may fail to capture the complete context information of each area. Conversely, pooling kernel size combinations with large intervals may lead to sparse features and overlook crucial context information. Although the PA of (1, 2, 4, 8) yielded superior results compared to (1, 2, 3, 6), the Dice and mIOU are more effective in representing the similarity between segmentation results and the ground truth. In Fig. 8, there are some unsatisfactory results in the three pooling combination segmentation methods. The combination of (1, 2, 3, 4) does not accurately segment thyroid nodules in certain images; however, under the combination of (1, 2, 3, 6), the segmentation of certain tracheal parts is incomplete. Finally, with the combination of (1, 2, 4, 8), there is an inaccurate segmentation of the nodes, an incomplete segmentation of the esophagus, and a misclassification of the carotid artery. After all, precise segmentation of nodules is more important in this task. So compared to others, the segmentation results under the combination of (1, 2, 3, 6) are better.

**Table 3:** Setting of pool kernel size of feature pyramid

Num	kernel size	Dice	mIOU	PA
1	1, 2, 3, 4	0.8053	0.7767	0.9439
2	1, 2, 3, 6	**0.8087**	**0.7887**	0.9434
3	1, 2, 4, 8	0.8021	0.7770	**0.9446**

The Dice (dice coefficient) is a set similarity measurement function, which is usually used to calculate the similarity between two sets. You can see in Eq. 14

The IOU (intersection over union) is the ratio of the intersection and union of the predicted result of a certain category and the true label. You can see in Eq. 15

For multi-category semantic segmentation, the average intersection over union ratio mean IOU (mIOU) is generally used as the evaluation indicator, that is, the IOU of each category is summed and then averaged

The PA (pixel accuracy) is the percentage of correct predicted pixels in the total number of pixels. You can see in Eq. 16

The CPA (category pixel accuracy) is the percentage of pixels whose real tags also belong to category. You can see in Eq. 17

Therefore, by combining three segmentation evaluation indicators with actual segmentation results and combining quantitative and qualitative analysis, the pooling kernel of the pyramid feature module is set to (1, 2, 3, 6) in this task.

After the experiment of setting the pooling kernel size of the PPM module and selecting the decoding method, the ablation experiment combined with each module is carried out. Taking the basic U-net++ network as the baseline, the PPM module and Attention gating module are introduced, respectively, and their joint experiments are compared. 300 epochs are trained on the model, and Dice coefficient, mIOU, and PA are evaluated.

**Fig. 9:**
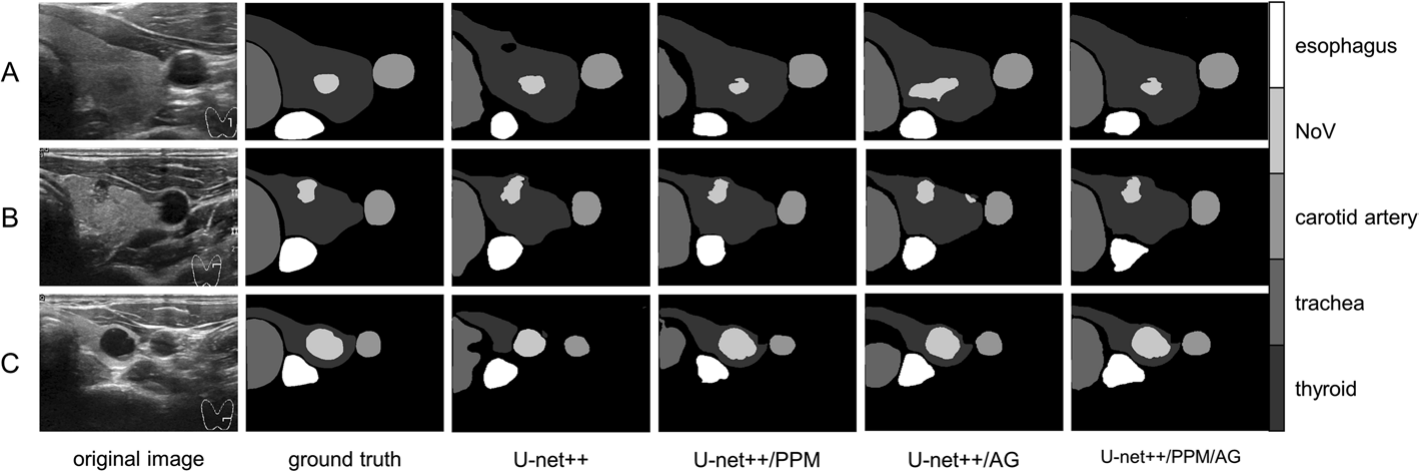
Segmentation results with different algorithm combinations

As shown in Table 4**,** based on U-net++, put PPM, and AG into the framework, respectively. The three evaluation indexes have increased which is compared with U-net++ as the baseline. Furthermore, the U-net++ combined with PPM and AG presents a more excellent effect, in which the best Dice get 0.8188, the mIOU notch up 0.8035 and the PA achieve 0.9479. Based on the results of U-net++, the Dice rise 3.10%, the mIOU swell 6.91%, and the PA index gain 1.84%. All evaluation indicators were statistically analyzed and p-value and p < 0.05 were calculated. Displayed in Fig. 9, as a whole, the segmentation results from U-net++ are used as a baseline, and different algorithmic improvements are introduced, namely the pyramidal feature pool and the attention gating mechanism described here. Whether used alone or in combination, has achieved certain improvements in segmentation performance, but the degree of improvement varies. The segmentation effect of U-net+/PPM/AG is significantly improved compared to the first three models. The segmentation of thyroid entities, nodular lesions (including thyroid internal blood vessels), and esophagus is better, and particularly, the first three models did not achieve good segmentation results for the trachea part. In this model, significant improvements were achieved, with clear boundaries and relatively complete shapes for the tracheal part. PPM can fully expand the network receptive field, integrate the context information of different regions, and improve the network's ability to obtain global information. AG can combine characteristic layers and perform weighting calculations. In this way, the network can pay more attention to the segmented target structure and suppress the background which is the irrelevant areas. Thereupon, the deep supervision algorithm is combined to capture the spatial location information of the organizational structure and maximize the global information. These are of great significance for semantic segmentation.

**Table 4:** The impact of different algorithms on the network model

Num	Tricks	Dice	mIOU	PA
1	U-net++	0.7878	0.7344	0.9295
2	U-net++/PPM	0.8087	0.7887	0.9434
3	U-net++/AG	0.8082	0.7847	0.9458
4	U-net++/PPM/AG	**0.8188**	**0.8035**	**0.9479**

The Dice (dice coefficient) is a set similarity measurement function, which is usually used to calculate the similarity between two sets. You can see in Eq. 14

The IOU (intersection over union) is the ratio of the intersection and union of the predicted result of a certain category and the true label. You can see in Eq. 15

For multi-category semantic segmentation, the average intersection over union ratio mean IOU (mIOU) is generally used as the evaluation indicator, that is, the IOU of each category is summed and then averaged

The PA (pixel accuracy) is the percentage of correct predicted pixels in the total number of pixels. You can see in Eq. 16

The CPA (category pixel accuracy) is the percentage of pixels whose real tags also belong to category. You can see in Eq. 17

After the improved U-net++ network model is finally determined, the joint use of different algorithms and the corresponding super parameter settings are completed, complete the training of the model and evaluate the model with the test set. The CPA of each segmentation part is calculated and evaluated, and the results are shown in Table 4:

As shown in Table 5, in general, the CPA index of each organization structure performs well, with all of them receiving a score that surpasses 0.8500. This segmentation is sufficient for 3D reconstruction.

**Table 5:** Segmentation accuracy of different parts

Num	Name	CPA
1	Background	0.9535
2	Thyroid	0.9488
3	Trachea	0.9056
4	NoV	0.8625
5	Esophagus	0.9219
6	Carotid artery	0.9424

**Fig. 10:**
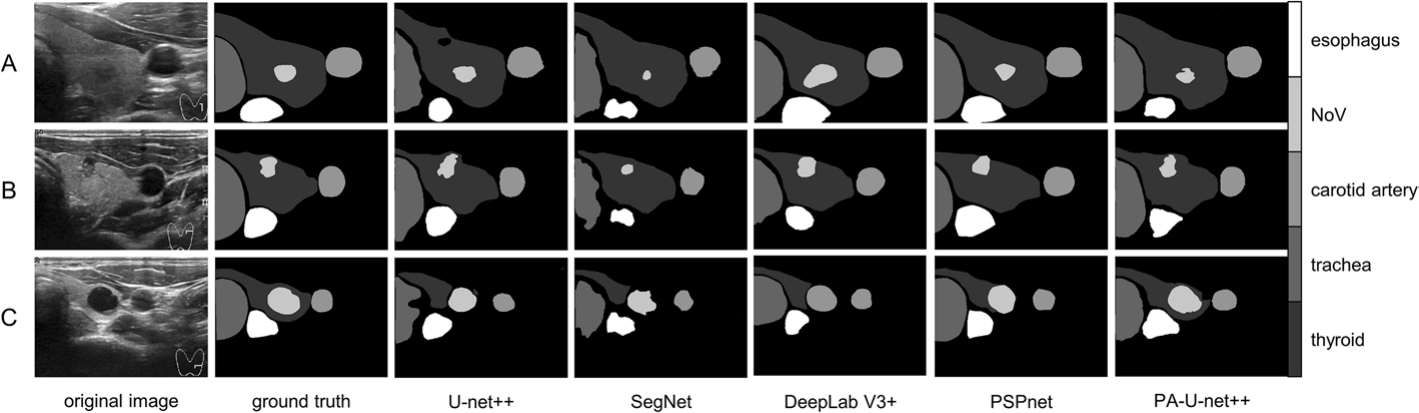
Effectiveness of different models for segmentation of multiple tissues of the thyroid glands

And not only comparing the segmentation results with U-net++, this paper also compares them with state-of-the-art models that are currently commonly used in the segmentation field. The segmentation results obtained were compared under the same experimental setup, and the trainable parameters of different models are shown in Table 6. All evaluation indicators were statistically analyzed and p-value were calculated (*p* < 0.05). The PA-U-net++ proposed in this paper performs optimally in Dice and mIOU, and PSPnet gets the best PA index. And Dice and mIOU can better evaluate segmentation effects in semantic segmentation tasks. So, PA-U-net++ has a better performance in this task. Although the number of trainable parameters in the model proposed in this paper is much larger than that of the basic U-Net++, it still has fewer parameters compared to other advanced segmentation models. The improved effect of the segmentation also proves the feasibility of increasing the parameters. The segmentation results of U-net++, SegNet, DeepLabV3+, PSPnet and this paper's method PA-U-net++ are shown in Fig. 10.

**Table 6:** The evaluation of segmentation results for different models

Num	Models	Dice	mIOU	PA	Parameters(M)
1	U-net++	0.7878	0.7344	0.9295	9.16
2	SegNet	0.7233	0.7026	0.9151	29.45
3	DeepLabV3+	0.7743	0.7175	0.9236	26.72
4	PSPnet	0.8030	0.7864	**0.9495**	32.23
5	PA-U-net++	**0.8188**	**0.8035**	0.9479	25.24

The Dice (dice coefficient) is a set similarity measurement function, which is usually used to calculate the similarity between two sets. You can see in Eq. 14

The IOU (intersection over union) is the ratio of the intersection and union of the predicted result of a certain category and the true label. You can see in Eq. 15

For multi-category semantic segmentation, the average intersection over union ratio mean IOU (mIOU) is generally used as the evaluation indicator, that is, the IOU of each category is summed and then averaged

The PA (pixel accuracy) is the percentage of correct predicted pixels in the total number of pixels. You can see in Eq. 16

The CPA (category pixel accuracy) is the percentage of pixels whose real tags also belong to category. You can see in Eq. 17

### The results of 3D visualization

**Fig. 11:**
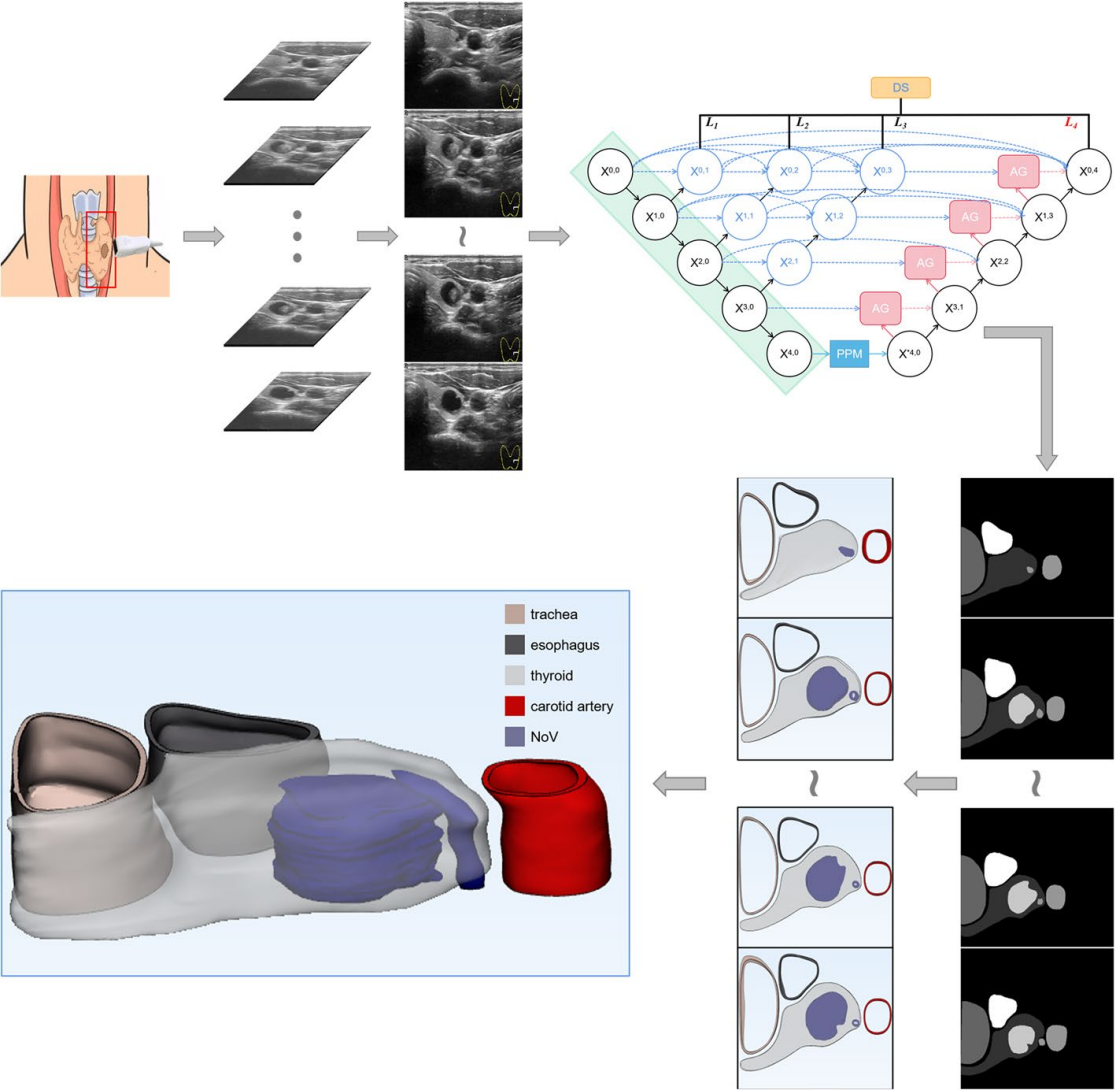
Processes for 3D visualization of multi-organizational structures

**Fig. 12:**
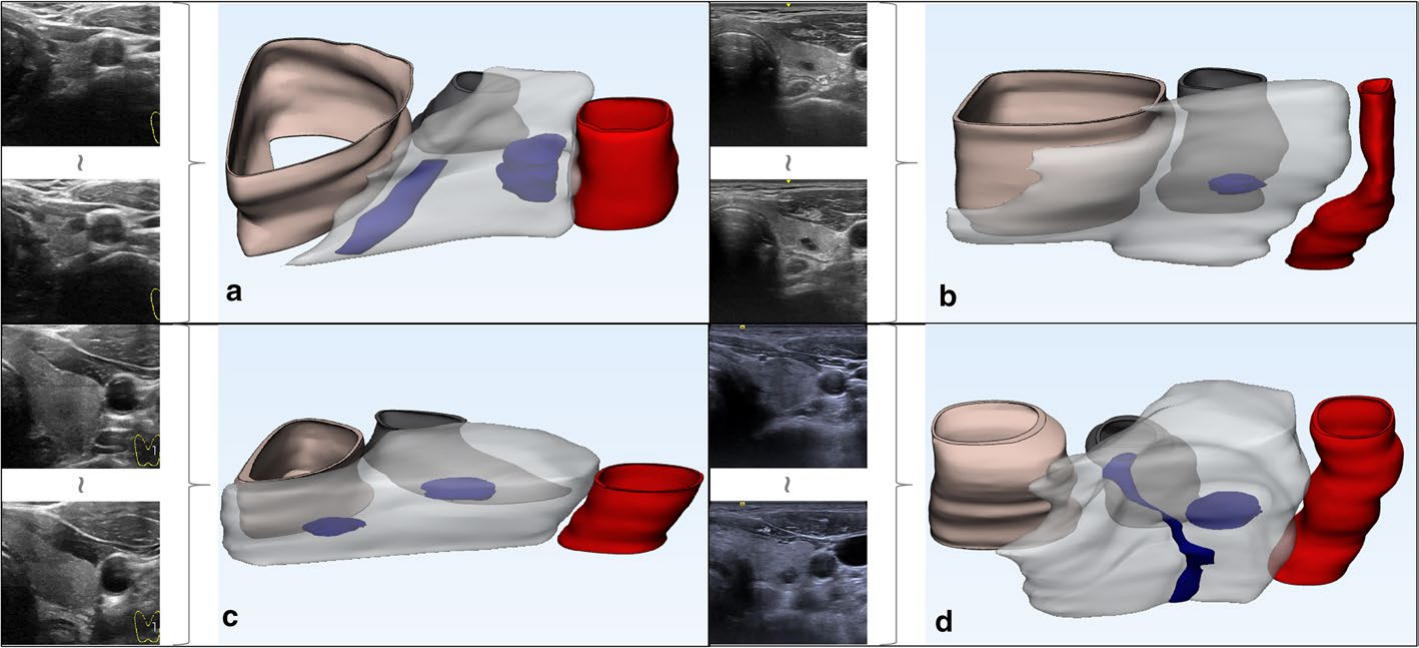
The three-dimensional visualization of four cases of thyroid nodules

As shown in Fig. 11, ultrasound images of the thyroid gland acquired by the ultrasound probe are fed into PA-Unet++ for multi-tissue segmentation and the results are used for 3D visualization. As shown in Fig. 12, it clearly shows the spatial relationship of each organizational structure and its own spatial representation. It can accurately distinguish the nodule lesions inside the thyroid gland from the interpenetrating blood vessels. Both single nodular lesions and multinodular lesions can be well demonstrated. In addition, if the patient has other tissue invasion lesions such as esophageal diverticulum, it can also be clearly distinguished from thyroid nodules by three-dimensional visualization results. In this way, it can reduce the misdiagnosis of nodules. Moreover, the intuitive spatial location information can serve for treatment planning and surgical navigation.

## Conclusion

The 3D visualization for thyroid ultrasound images is unsatisfactory, the root cause being poor multi-target segmentation. This paper proposed a novel method for automatic Multi-tissue segmentation of ultrasound thyroid scanning video using an improved U-net++ model. The nodules and vessels within the thyroid gland were considered as a class for segmentation. Then, the 3D reconstruction results were used to present spatial information to differentiate the nodules from the internal blood vessels in terms of positional relationships and spatial representations. In addition, other tissues around the thyroid gland are also segmented and reconstructed to show the relationship between the tissues more intuitively. This can also help in the diagnosis of invasive lesions in the peri-thyroid tissue (e.g., esophageal diverticula) and avoid confusion with nodular lesions.

In this study, PA-Unet++ improves upon the U-net++ architecture by incorporating the pyramid pooling module (PPM) and attention gating (AG). Our evaluation results demonstrate that this method can accurately segment the thyroid gland, thyroid nodule (including thyroid internal blood vessels) and surrounding tissue structure, and reconstruct them in three dimensions. The 3D visualization results in a clearer distinction between thyroid nodules and invasive lesions of blood vessels and other tissues within the thyroid gland, leading to a more precise diagnosis of neck disorders. Moreover, the intuitive spatial location information can serve for treatment planning and surgical navigation.

## Discussion

The PA-U-net++ proposed in this paper is able to perform multi-target segmentation of thyroid nodules and their surrounding tissue structures on ultrasound thyroid images, which improves the effectiveness of thyroid multi-target segmentation to a certain extent. The medical image data come from the clinic, but the difficulty in obtaining standard available thyroid ultrasound video data leads to less available data for multi-target segmentation, which limits the optimization of model training. Secondly, the algorithmic model was not really applied to the clinic and no clinical validation was performed to test its real effect. Therefore, it is subsequently hoped that more available data can be acquired and the algorithmic model can be applied to clinical practice to verify its performance.

## Data Availability

Data and the programming code used as part of this research can be obtained from authors on a request.
